# Distribution of Human Papillomavirus and Antisperm Antibody in Semen and Its Association with Semen Parameters Among Infertile Men

**Published:** 2020

**Authors:** Ahmad Piroozmand, Seyed Dawood Mousavi Nasab, Mahzad Erami, Seyed Mohammad Ali Hashemi, Elnaz Khodabakhsh, Nayebali Ahmadi, Zahra Vahedpoor

**Affiliations:** 1- Department of Microbiology and Immunology, School of Medicine, Kashan University of Medical Sciences, Kashan, Iran; 2- Autoimmune Diseases Research Center, Kashan University of Medical Sciences, Kashan, Iran; 3- Department of Research and Development, Production and Research Complex, Pasteur Institute of Iran, Tehran, Iran; 4- Beheshti Hospital, Kashan University of Medical Sciences, Kashan, Iran; 5- Department of Virology, School of Medicine, Golestan University of Medical Sciences, Gorgan, Iran; 6- Infertility Clinic, Shahid Beheshti Hospital, Kashan University of Medical Sciences, Kashan, Iran; 7- Proteomics Research Center, Shahid Beheshti University of Medical Sciences, Tehran, Iran; 8- Obstetrics and Gynecology Department, Kashan University of Medical Sciences, Kashan, Iran

**Keywords:** Antisperm antibody, HPV, Male infertility, Semen parameter

## Abstract

**Background:**

Sexually transmitted infections (STIs) can be associated with infertility. Human papillomavirus (HPV) has been identified as a potential agent in male infertility. Also, anti-sperm antibodies (ASA) have been detected in men with infertility. The aim of this study was to investigate the prevalence and association of HPV and ASA in infected semen of infertile men.

**Methods:**

This cross-sectional study was performed on 96 infertile men referring to infertility treatment center of Kashan University of Medical Sciences during March 2017 till September 2017 in Iran. Semen analysis and diagnostic PCR test were performed for detection of HPV DNA. The semen parameters in HPV infected and ASA positive samples were compared with HPV non-infected and ASA negative samples. Chi square test was used to determine the correlation between variables and p<0.05 was considered statistically significant.

**Results:**

HPV DNA and ASA were detected in 17.4% and 15.2% of 96 semen samples, respectively. Semen volume, sperm count, sperm motility and the normal morphology rate were significantly decreased in HPV-positive subjects (p=0.004, p= 0.016, p<0.001, and p=0.017, respectively). Also, sperm motility was significantly decreased in ASA-positive subjects (p=0.002), also patients with HPV infection had a higher rate of ASA than the non-HPV group. In contrast to ASA, HPV infection had a significant correlation with education level (p=0.039).

**Conclusion:**

The findings suggest that asymptomatic seminal infection of HPV and ASA by adversely affecting sperm quality, in particular sperm motility and count, may play an important role in male infertility.

## Introduction

Infertility refers to the inability to have a child after at least one year of unprotected sex (1). Infertile couples suffer mainly from primary infertility across the world (2). Male infertility is found in at least 40% of couples referring for treatment. Number, motility and morphology of sperm are the main indicators of sperm fertility, so that the appearance of sperm is the best predictor for pregnancy (3). Semen infection is regarded as an important factor in infertility of asymptomatic men, as the quality of semen is decreased in these individuals. Evidence suggests that some of the viral infection can lead to male infertility, directly or indirectly, causing local infections and immunological responses, which in turn can have a negative impact on reproduction (4–6). Sexually Transmitted Infections (STDs) represent a preventable cause of infertility around the world. Several viruses, including Epstein-Barr virus, hepatitis B virus, cytomegalovirus, human papillomavirus, Herpes simplex virus type 2, human herpes virus type 6, HIV type 1 and hepatitis C virus have been detected in semen from asymptomatic men (6–8). One of these viral infections is human papilloma virus (HPV). Contrary to the clinical importance of HPV in the development of lower genital tract carcinoma, little attention has been paid to the transmission of HPV through semen (5). The presence of HPV in semen has been reported, but its effect on sperm parameters is negligible (9, 10). The presence of HPV in semen samples is associated with a decrease in sperm motility, suggesting the important role of HPV in male infertility (11). Moreover, many authors have suggested that HPV can cause changes in pH of semen and fragmentation of the spermatozoa DNA (12, 13). Despite new therapies for infertility treatment, a number of couples have not been cured yet. Various reports about the relation between HPV and Anti-Sperm Antibody (ASA) and infertility in men exist, but so far no similar study has been conducted on the relationship between the two variables of HPV and ASA. Thus, in this study, an attempt was made to investigate frequency of HPV infection and ASA in semen samples and its association with sperm parameters among infertile men of Kashan, Iran.

## Methods

### Study population and semen processing:

This cross-sectional study was performed on 96 infertile men referring to Kashan University of Medical Sciences infertility treatment center located in Kashan, Iran, during March 2017 to September 2017. The semen samples of infertile men were collected after 48 to 72 *hr* of sexual abstinence prior to sampling and the subjects didn’t take antibiotic during the last one week. None of the patients had clinical symptoms of genital herpes and genital warts. After liquefaction at room temperature, semen volume, pH, sperm count, viability, motility, and normal morphology were determined according to World Health Organization guidelines for semen analysis (14). The protocol of the present study was approved by the Ethics Committee of Kashan University of Medical Sciences and written informed consent forms were signed by all subjects.

### DNA extraction:

Two-hundred microliters of the sample was centrifuged at 2500 *rpm* for 15 *min*. Supernatant was removed and the pellet was used for the extraction by the Genomic DNA Extraction Kit (BIONEER, South Korea) according to the protocol.

### HPV DNA detection:

After DNA extraction, using general screening primers and then with the specific primers of HPV18 E6 and HPV16 E7, HPV detection and genotyping were performed (Table 1) according to previous studies (15, 16). The final volume of PCR reaction was 30 *μl* and each reaction contained 5.5 *μl* of 2x master mix (Bioneer’s AccuPower PCR PreMix, Korea), 3 *μl* of DNA, 0.5 *μl* of Taq DNA polymerase (CinnaGen, Iran), 0.5 *μl* of each primer and 20 *μl* of DEPC water. Amplification cycles were set as follows: for HPV16, 95°*C* for 30 *s*, 57°*C* for 45 *s*, 72°*C* for 60 *s* and 72°*C* for 5 *min*; for HPV18, 95°*C* for 30 *s*, 55°*C* for 45 *s*, 72°*C* for 60 *s* and 72°*C* for 5 *min*.

**Table 1.** HPV detection and genotyping primers

**Primer**	**Amplicon size (*bp*)**	**Sequence**	**Ref**
**HPV (MY09/MY11)**	540	F-CGTCC(AC)A(AG)(AG)GGA(T)ACTGATC-3 R-GC(AC)CAGGG(AT)CATAA(CT)AATGG-3	[16, 17 ]
**HPV18 E6**	208	F: 5-CGTCC(AC)A(AG)(AG)GGA(T)ACTGATC-3 R: 5-GC(AC)CAGGG(AT)CATAA(CT)AATGG-3	[18]
**HPV16 E7**	196	F: 5-GTCTACGTGTGTGCTTTGTACGCAC- 3 R: 5-ATATATGTTAGATTTGCAACCAGAGACAAC- 3	[18]

### ASA detection:

The detection of IgG anti-sperm antibody in each semen sample was performed using ELISA kit (Greiner, Germany). All processes were performed according to the manufacturer’s protocol.

### Statistical analysis:

After determining the frequencies, Chi square test was used to determine the correlation between variables. The p<0.05 was considered significant. In this study, SPSS soft-ware version 17 was employed for statistical analysis.

## Results

**Figure 1.**
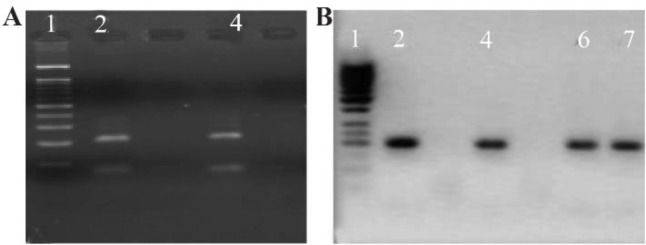
Detection of HPV genotype by PCR in semen cases. Gel figure A shows the presence of HPV 18 infection with an amplicon of E6 consensus (208 *bp*) and gel figure B represents the amplicon of HPV 16 E7 (196 *bp*). A: Results of PCR HPV 18 genotype. Lines 2 and 4 were HPV 18 positive. B: 2, 4, 6 and 7 cases are positive for HPV 16. Molecular weight marker for A is 100 *bp* and for B is 50 *bp*

In this study, ninety six infertile men were enrolled. The mean (SD) age was 32.15±6.66 years. The mean semen volume (SD) was 3.56±1.47 *ml*, the mean total number of sperm (SD)×10^6^ was 64.11±32.39, the mean pH=7.80±0.5 and the average duration of infertility (SD) was 3.92±4.33 years. The findings of this study showed that viral infection rates of HPV and distribution of ASA in infertile men was 16(17.4%) and 14(15.2%), respectively. Of 16 positive cases for HPV, eight persons were infected with HPV 16, four cases with HPV 18 and HPV 16/18 co-infection was detected in four patients (Figure 1).

In addition, HPV infection and ASA infection were not associated with age (p=0.608), duration of infertility (p=0.865) and pH (p=0.843); the means are shown in table 2. In contrast to ASA, HPV infection had a significant correlation with education level (p=0.039).

**Table 2.** The association between HPV and ASA with mean of infertility period, pH and age

**Variable**	**HPV (%)**			**ASA (%)**		

	**Positives**	**Negative**	**p-value**	**Positives**	**Negative**	**p-value**
**Infertility period (year)**	3.64	3.99	0.762	3.75	3.95	0.865
**pH**	7.80	7.80	0.826	7.80	7.80	0.843
**Age (year)**	33.06	31.96	0.551	33.00	32.00	0.608

The frequency of semen volume (p=0.004), total sperm count (p=0.016), morphology (p=0.017), and sperm motility (p<0.001) were significantly associated with HPV (Table 3). Additionally, sperm motility was significantly associated (p=0.002) with ASA (Table 3).

**Table 3.** The prevalence of HPV based on sperm quality parameters in infertile men

**Parameters**	**HPV positive**	**HPV negative**	**p-value**	**ASA positive**	**ASA negative**	**p-value**
**Sperm volume**						
Normal	17 (89.5%)	77 (100%)	0.004	15 (94%)	79 (99%)	0.201
Abnormal	2 (10.5%)	0 (0%)		1 (6%)	1 (1%)	
**Sperm count**						
Normal	7 (37%)	51 (66%)	0.016	9 (56%)	49 (62%)	0.666
Abnormal	12 (63%)	25 (34%)		7 (44%)	30 (38%)	
**Sperm morphology**						
Normal	10 (53%)	63 (82%)	0.017	10 (67%)	63 (79%)	0.309
Abnormal	8 (47%)	14 (18%)		5 (33%)	17 (21%)	
**WBC**						
Normal	0 (0%)	0 (0%)	-	0 (0%)	0 (0%)	-
Abnormal	19 (100%)	77 (100%)		16 (100%)	80 (100%)	
**Sperm motility**						
Normal	1 (5%)	39 (51%)	<0.001	2.5% (1)	39 (49%)	0.002
Abnormal	18 (95%)	38 (49%)		26.8% (15)	41 (51%)	
**Viscosity**						
Normal	19 (100%)	71 (92%)	0.209	15 (94%)	75 (94%)	0.999
ST	0 (0%)	6 (8%)		1 (6%)	5 (6%)	
**Color**						
M	19 (100%)	78.7% (70)	0.394	16 (100%)	73 (91%)	0.470
LY	0 (0%)	100% (5)		0 (0%)	5 (6%)	
YT	0 (0%)	100% (2)		0 (0%)	2 (3%)	

M: Milky, LY: Light yellow, YT: Yellow turbidity. Parametric tests such as t-test or ANOVA were used to determine the association and Mann-Whitney, Fisher’s Exact test and Chi Square test were used for abnormal distribution

## Discussion

The results of this study showed that HPV infection was positive in 17.4% of infertile men and there is a significant relationship between HPV and sperm quality. Until now, widespread studies have surveyed HPV prevalence in infertile men that ranged from 10 to 30% in the world as well as Iran (13, 16–19). Meanwhile, the previous data from a study in Mexico (20) revealed the prevalence of HPV in semen (59.73%) of the men. This amount was higher than the findings of our study. In a research conducted by Yang et al. (2013) in China, 17.4% of infertile men were positive for HPV (12). In a study carried out by Luttmer et al. in Netherlands (2015), the prevalence of HPV in the semen of infertile men was 14.9% (22). In the study conducted by Moghimi et al. of the infertile patients, HPV infection prevalence was 11.43%, which was lower than that obtained in our study (17.4%). An imaginable explanation for the difference between these findings and the results of our study is the differences in the prevalence and distribution of HPV based on geographical areas, demographic and social characteristics, behavioral factors and lifestyle, sexual behaviors among infertile men and types of techniques used in every study (24–26). Some STIs such as chlamydia, mycoplasma, and HPV damage semen quality by inducing epididymitis, orchitis or urethritis (5). Among different parameters of sperm quality in the present study, total sperm count, morphology and sperm motility were significantly correlated with HPV infection. Our results showed that compared with HPV-negative men, the rate of normal sperm morphology significantly decreased in HPV- positive cases. The various studies reported different findings from sperm morphology between HPV-infected and uninfected men. Moghimi et al. and Yang et al. found that abnormal morphology of sperm clearly increased the number of HPV-infected individuals (12, 23). High-risk HPV16 was predominant in male anogenital sites, prostate, bladder and oropharynx (27, 28). Our study indicated that HPV16 was the most common type in semen, accounting for approximately one-fifth of HPV-positive samples that is similar to the study that reported HPV 16 is more prevalent than HPV 18 in Iranian cases (18). Moreover, Luttmer showed that between HPV types, HPV 16 is frequently present in semen (29).

The relationship between HPV and its negative effect on sperm morphology remains weakly understood, but it could be due to binding of HPV to the spermatozoa head (30). Like previous studies, the majority of our finding showed reduced sperm motility in men infected with HPV (31). In contrast, a few studies have reported enhanced motility and progression in HPV-exposed sperm (32) and some of the studies have shown no association between HPV infection and sperm quality parameters (33).

In this study, high-risk HPV infection did significantly affect total sperm count in infertile men. The studies reported a significant relationship between the lower total sperm count and HPV infection in semen (5). In this study, also HPV infection did significantly affect total sperm count in infertile men. Unlike our result, Rintala et al. (34) and Moghimi et al. (23) reported that HPV decreased sperm counts in infertile Iranian men. The antisperm antibodies are the cause of sub-fertility in 5–15% of male patients and cause of infertility in 1–2% of men (35, 36). ASA was found to be positive in 15.2% of infertile men. Among the different parameters of sperm quality investigated in the presence of ASA in semen, only sperm motility was significantly decreased in ASA-positive subjects (p=0.002). Also, our results are similar to some previous reports suggesting that ASA is generally associated with poor sperm motility, and reduced natural pregnancy rates (37, 38) and similar to Cui et al.’s study, sperm concentrations and sperm abnormal morphology show non-significant difference (39). The current study demonstrates that patients with HPV infection had a higher rate of ASA than the non-HPV group. These findings suggested that the presence of HPV on the sperm surface may lead to an antigenic stimulation for the formation of ASA.

The results of this study showed that HPV infection and ASA were not associated with age, pH and duration of infertility. In contrast to ASA, HPV infection had a significant correlation with the education level (p<0.039). This result is in accordance with previous studies (22, 23). Perino et al. reported that the presence of HPV in semen was associated with reduced fertility and the rate of abortions increased when using Assisted Reproductive Technology (ART) (40). In contrast, some studies reported no effect on the quality of sperm (31).

## Conclusion

HPV infection and antisperm antibody via the effect on total sperm count, morphology and sperm motility, decrease sperm quality and result in male infertility. Also, these results suggest that young couples can be tested for HPV and ASA along with other causes of infertility.
